# The Effect of Group Composition and Mineral Supplementation during Rearing on Measures of Cartilage Condition and Bone Mineral Density in Replacement Gilts

**DOI:** 10.3390/ani9090637

**Published:** 2019-08-30

**Authors:** Phoebe Hartnett, Laura Boyle, Bridget Younge, Keelin O’Driscoll

**Affiliations:** 1 Teagasc Pig Development Department, Animal & Grassland Research and Innovation Centre, Moorepark, Fermoy, Co. Cork P61 P302, Ireland; 2 Department of Biological Sciences, University of Limerick, Limerick V94 T9PX, Ireland

**Keywords:** lameness, osteochondrosis, cartilage lesions, welfare, gilt, nutrition, cartilage, health

## Abstract

**Simple Summary:**

The lifetime performance of commercial sows relies on longevity, which is dependent on good health, particularly, limb health. In many countries, young female pigs (gilts) intended for breeding are often reared with male finisher pigs destined for meat production. However, finisher diets are not designed to meet the needs of developing gilts and may not supply the necessary minerals to support good limb health. Moreover, gilts reared with uncastrated (i.e., entire) male pigs are exposed to high levels of sexual mounting and aggression, which may cause physical damage. This experiment investigated the effect of female-only or mixed-sex rearing with and without supplementary minerals (Copper, Zinc and Manganese) on locomotory ability, cartilage condition and areal bone mineral density (aBMD) of breeding age gilts. The addition of the minerals to the diet resulted in increased aBMD in the humerus bone compared to gilts on the control diet. Rearing gilts in female-only groups reduced the number of cartilage lesions, and there were fewer incidences of elbow cartilage fractures. Overall, both strategies (mineral supplementation and female-only rearing) had benefits for limb health, which could help to improve sow longevity.

**Abstract:**

Lameness is a major cause of poor longevity and poor welfare in replacement gilts. The problem is exacerbated by inappropriate housing and diet during the rearing period. Replacement gilts are often reared with male finisher pigs destined for slaughter. If they are not castrated, they perform high levels of potentially injurious sexual and aggressive behaviour. Furthermore, finisher pig diets are not designed to meet the needs of developing gilts and may not supply the necessary minerals to support good limb health. The objective of this study was to evaluate the effect of supplementing the diet of replacement gilts with copper, zinc and manganese and separating them from males during the rearing period on locomotory ability, bone mineral density and cartilage lesion scores. A 2 × 2 factorial design experiment investigated the effect of female-only or mixed-sex rearing, with or without supplementary minerals (Copper, Zinc and Manganese). In total, 384 maternal line gilts were assigned to 32 pens of 12 and were locomotion scored during the rearing period. A sub-sample (n = 102) of gilts were culled at breeding age and the front right limb was removed at slaughter. Areal bone mineral density (aBMD) was measured using dual energy X-ray absorptiometry, after which the limb was dissected to score the condition of the cartilage. The addition of trace minerals to the diet resulted in increased aBMD in the humerus (*P* < 0.05) compared to the control diet. Rearing gilts in female-only groups reduced the number of cartilage lesions overall (*P* < 0.05), and on the humeral condyle (*P* < 0.05). Rearing replacement gilts in female-only groups and with mineral supplementation had benefits for limb health.

## 1. Introduction

Lameness in pigs is a significant welfare issue [1] and is a major reason for poor longevity in sows [2,3]. In general, locomotory issues account for 13% of all sow cullings, and over half of these females have not attained their second parity [4]. The aetiology of lameness in pigs is multifactorial and risk factors include flooring—concrete flooring in general and particularly slatted concrete [5], stocking density and pen size [6], housing type, growth rates and nutrition [7]. In recent years, there have been interest in the way replacement gilts destined to enter the breeding herd are reared as a risk factor for lameness [8].

In many countries, replacement gilts are reared similarly to finisher pigs and in mixed-sex groups up to breeding age [7,9]. This means that in countries that do not practice castration (e.g., Ireland) replacement gilts are kept with entire male pigs which perform high levels of aggressive and mounting behaviour [10,11]. These behaviours increase the risk of injuries, such as limb and cartilage damage [12], which are associated with lameness.

Additionally, diets designed for finisher pigs may not meet the physiological needs of the replacement gilt [8]. In particular, such diets may not supply the correct balance of minerals to satisfy the nutritional requirements for reproductive performance, cartilage and bone formation and integrity [13,14,15]. There are few established guidelines with regard to trace mineral requirement, and often, gilt diets are developed using levels appropriate for finisher pigs, or gestating sows; in fact, there are no trace mineral recommendations for developing gilts in the most recent edition of the Nutritional Requirements for Swine [16]. Nevertheless, Ferket [17] reported that copper, zinc and manganese reduced lameness and leg problems in sows. Zinc is particularly important for claw horn production due to its vital role in cell repair and replacement [18,19,20]. Manganese plays a vital role in horn production and the formation and maintenance of cartilage and bone; in cattle deficiency, it is associated with short/weak bones [18,20]. Minerals can play other important roles in promoting sow longevity. For example, copper is essential for antibody development and lymphocyte replication [21]. Studies with pregnant sows found that the addition of these trace minerals to the diet improved claw health [22] and reduced the prevalence of lameness and leg abnormalities [17]. A similar study reported improved locomotion scores, higher bone mineral density and lower cartilage lesion scores in gilts fed a restricted diet formulated for fat rather than lean deposition with increased levels of copper, zinc and manganese [8]. However, the study used terminal line gilts, and furthermore, restricted feeding is difficult to conduct in practice, as most maternal line gilts are reared in finisher pig accommodation. Hence, we hypothesised that supplementing a standard finisher ad lib fed diet with copper, zinc and manganese and separating gilts from male pigs during the rearing period would result in improved locomotory ability, higher bone mineral density and lower cartilage lesion scores.

In addition, previously used joint scoring methods, which we found in the literature, are relatively subjective in nature (e.g., 1 = normal to 4 = severe abnormality [8]). Thus, with the aim of capturing more detail on the nature and amount of damage to the joint cartilage and improve the objectivity of the scoring system, we also aimed to develop an improved method of scoring joint surface lesions and compared this to a previously published version.

## 2. Materials and Methods

This study was carried out in the 200-sow unit at the Pig Development Department in Moorepark, Fermoy, Co. Cork, Ireland, between March and August 2017. The experimental work was authorized by the Teagasc animal ethics committee (Approval no.: TAEC136-2016) and licensed by the Health Products Regulatory Authority (License no: AE19132-P057).

In August 2016, 52 sows in the Moorepark herd were served using maternal line semen from Landrace sires (0153H Longo and 0096H Grande from Hermitage Pedigree Pigs Ltd., The Hermitage, Sion Road, Kilkenny, Ireland). Four batches of sows were served to create four replicates, each three weeks apart (average 13 sows per replicate). At farrowing, these sows provided a pool of 677 piglets for use in the experiment. The experimental period was from December 2016 (weaning of the first replicate, d27.4 ± 0.4) to August 2017 (breeding age of the last replicate, d196.3 ± 0.5).

All the piglets were weighed and tagged at birth, then back tested at 21.6 ± 2.38 days of age. Each piglet was lifted out the pen and placed on its back on a V board, then held in place by the experimenter. The right hand was placed on the piglet’s thorax with the forefinger between the front legs, and the hind legs were held using the other hand. The test started when the piglet remained still for 3 seconds and lasted for exactly 60 seconds, which was determined using a stop-watch. The number of resistance attempts (struggles) was counted. At weaning (27.4 ± 0.4 days of age), pigs were weighed, then blocked on the basis of sow, sire, sex, back-test score, and wean weight, and assigned to one of 32 (8 pens per treatment) separate groups of 12 pigs (96 ± 16.9 pigs per replicate). Each pen had an even distribution of sire (Longo or Grande) and back test score (av.2, range 0–5), and a wean weight average of 7.8 kg ± 1.5. Half of the groups contained only females (FEM) and half of the groups were mixed (MIX; 6 males, 6 females). Four focal gilts were selected from each pen, according to their back-test scores; one was a low responder (score 0–1), two were picked as medium responders (score 2–3) and one was a high responder (4–5). Any piglet with a score of 5+ was not included in the trial.

Pigs were assigned to their treatment groups using a 2 × 2 factorial design with the first factor being group composition (FEM or MIX as described above) and the second factor being mineral supplementation. The control diet (control; CON) represented a standard finisher diet used on commercial pig farms. This was fed to all pigs from entry to the finisher stage d81.3 ± 0.5 until d117.5 ± 0.6 (16.8 ± 0.1 wks) of age when pigs in half of the groups were switched to a similar diet, but supplemented (SUPP) with Availa®Sow minerals (Zinpro Corp, Eden Prairie, Minnesota USA; Table 1). This provided additional Copper, Zinc and Manganese (Table 2). All feed was in dry-pelleted form (3 mm).

The pigs were in the weaner stage accommodation for 8 weeks post weaning (i.e., d81.3 ± 0.5 of age), at which time they were moved to the finisher stage accommodation. They remained in the finisher accommodation in the same groups for 10 weeks (i.e., d148.3 ± 8.0); at this point, half of their pen mates (all of the male pigs and 50% of the females) were sent for slaughter. The remaining 102 gilts stayed in the same pens (6 females per pen) until d196.3 ± 0.5 when they were sent for slaughter (weighing 143.6 ± 12.8 kg).

Both the weaner and finisher accommodation pigs had access to fresh water ad libitum from a drinker and to feed ad libitum from a single space trough feeder, which contained a second nipple drinker. Weaners were fed a starter feed for the first week, a link feed for the following two weeks, and thereafter, a standard weaner diet. The weaner pens (2.4 × 2.6 m) had plastic slatted flooring, and the finisher pens (4 × 2.4 m) had fully slatted concrete floors. The rooms used for the trial contained either 10 or 30 pens. All the rooms were mechanically ventilated. The temperature of the weaner rooms was kept at 27–28 °C for 1 week post weaning and reduced by 2 °C every 2 weeks. Heating was generated by hot water pipes (controlled via computer). Finisher rooms were maintained at 20 °C, with no heating, and mechanically ventilated. Artificial lighting was on from 07:00–17:00 h. There was also natural lighting through the windows. Light to darkness cycle was approximately 12:12 h to allow for normal circadian rhythm.

Environmental enrichment was provided in every pen at each production stage. Weaner pigs were provided with an EASYFIX LUNA 117 (Perssepark, Ballinasloe, Co. Galway, Ireland) floor toy and finisher pigs were provided with two sources of enrichment—a wooden post attached to the side of the pen by a plastic holder and a rubber chew toy (EASYFIX ASTRO 200 Perssepark, Ballinasloe, Co. Galway, Ireland) hanging overhead on chains. Pigs were inspected twice daily and sick/injured animals were treated immediately; all antibiotic treatments were recorded.

At slaughter, the right front leg was removed at the shoulder from all 102 carcasses in the chill room and frozen at −20 °C for further analysis.

For the purposes of this study, only locomotion scores from the 102 gilts that were slaughtered at breeding age and had their right front leg removed were considered. The gilts were locomotion scored every two weeks (14.1 ± 3.6 days) from d81.3 (i.e., the day they entered the finisher accommodation) until d165.8 of age (i.e., slaughter age). Locomotory ability was scored while the gilts walked on solid concrete for a distance of approximately 30 m, from the front, rear and side of the animal. All the observations were carried out by one trained observer. Locomotion was assessed using an adapted version of *Calderon-Diaz* [23] ranging from 0–5 (Table 3).

To determine areal bone mineral density (aBMD; g/cm^2^), the front right limb was scanned by dual energy X-ray absorptiometry (DEXA) using a Hologic QDR-4500 Elite bone densitometer and analysed using Apex software version 2.3.7. A total of 306 scans were performed on the 102 limbs collected from the factory. Three areas of each limb were scanned and analysed: the humerus (HUM), radius/ulna (RU) and metacarpal (MET). The scanning mode varied for the different parts of the limb; for the humerus and the metacarpal, the “lumber spine” analysis option was used, and for the radius/ulna, the “forearm” option was used. These scanning modes provided the best fit after carrying out test scans on each option for each part of the limb. For analysis of the HUM and RU, the limbs were placed on their side and positioned according to the scanning mode such that the laser was in the correct place (approximately over the elbow joint at the top of the limb). For analysis of the MET, the limb was positioned on a Styrofoam^™^ plinth as the leg needed to be positioned with the hoof (sole side down) flat on the table to ensure that the bones of the hoof did not overlap. The scans were analysed for bone mineral content (BMC; g) and BMD (g/cm^2^) for the total area of the specific bone, which was outlined using the software. Each leg was scanned from frozen. The scanner was calibrated every day using the software daily QC exam; a weekly QC was also carried out, which included a radiographic uniformity test.

**Figure 1:**
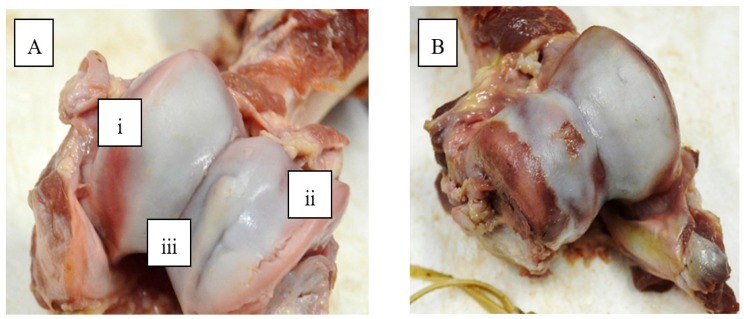
(**A**) (i) Thinning of cartilage, as evidenced by red bruise-like area (ii) Invagination of the cartilage and (iii) overgrowth, and (**B**) Osteochondrosis lesions (separation of cartilage from the underlying bone).

Dissection of the limb at the elbow joint was carried out to expose the humeral condyle (HC), which is made up of the capitulum and the trochlea and the trochlear notch (TN) surface. Two types of scoring methodology were used. First, the HC and TN were examined and given one score (Joint lesion score (JLS)) for any abnormalities present (i.e., invagination, thinning, overgrowth (Figure 1A) and fractures; cartilage score). The scoring system used was as per Quinn [8] and ranged from 1 (normal) to 4 (severe abnormality). The number of areas of osteochondrosis dissecans (OCD; detachment of the cartilage from the underlying bone (Figure 1B)) that were present were also counted (OCD prevalence).

The second scoring method was developed to capture more detail on the nature and amount of damage to the cartilage present. The number of areas of thinned cartilage (thinnings), invaginations and overgrowths on both the HC and TN were counted, as was the number of areas of OCD. All limb dissections and analyses were carried out by one trained observer.

Data were analysed using SAS v 9.4. The individual pig was used as the experimental unit. Locomotion scores were analysed using a generalised mixed model (PROC GLIMMIX) as the data were ordinal in nature. As there were extremely low numbers of pigs, which had a score of greater than 2, these were re-categorised as greater or equal than 2. A multinomial distribution was specified. Fixed effects included group composition (MIX v’s FEM), dietary treatment (CON v’s SUPP), and the interaction, as well as replicate. Pen was included as a random effect. The model could not converge when data from all weeks were included, and as such, each week was analysed separately, and p-values adjusted post-hoc using a bonferroni adjustment.

Areal bone mineral density (aBMD) was analysed using a general linear model (PROC MIXED), where the individual gilt was the experimental unit. Within each leg, each bone type was analysed separately.

The two different systems of cartilage scoring were initially compared, to determine whether the newly developed system was reflective of increases in damage, similarly to the previously published one. Pigs with cartilage which had at least one area of OCD were removed from the analysis. Analysis was carried out using a general linear model (PROC MIXED), with JLS as the fixed effect.

The JLS scores assigned to each limb using the method of [8] were analysed using generalised linear models (PROC GLIMMIX). Cartilage scores (1–4; multinomial distribution) were analysed separately to the number of areas of OCD (0, 1, 2 or 3; Poisson distribution).

For the second scoring methodology, the numbers of lesions were summed within lesion type and area of the cartilage (HC or TN). The overall sum for all cartilage areas, as well as the overall sum for the HC were analysed using linear mixed models. All other measures, other than overgrowths (incidence too low to be analysed statistically), were analysed using generalised linear mixed models. In instances when there were low numbers of individual scores, scores were combined with the next level on the scale (Table S1).

For all the models, fixed effects included group composition (MIX v’s FEM), dietary treatment (CON v’s SUPP), and the interaction, as well as replicate. Interactions were excluded from the model if found to be not significant. Cold weight was included in the models as a covariate. Pen was included as a random effect. In all the analyses where the mixed procedure was used, residuals were examined to verify normality and homogeneity of variances. Differences in least squares means were investigated using the t-test, followed by Tukey-Kramer adjustment for multiple comparisons. Degrees of freedom were estimated using Kenwood–Rogers adjustment. Statistical differences were considered significant at *P* ≤ 0.05. Tendencies towards significance (0.05 < *P* ≤ 0.10) are also presented. Data are presented as LSmeans and standard errors.

## 3. Results

### 3.1. Locomotory Ability

There was no effect of either group composition or mineral supplementation on locomotion score at any observation.

### 3.2. Areal Bone Mineral Density (aBMD)

**Figure 2:**
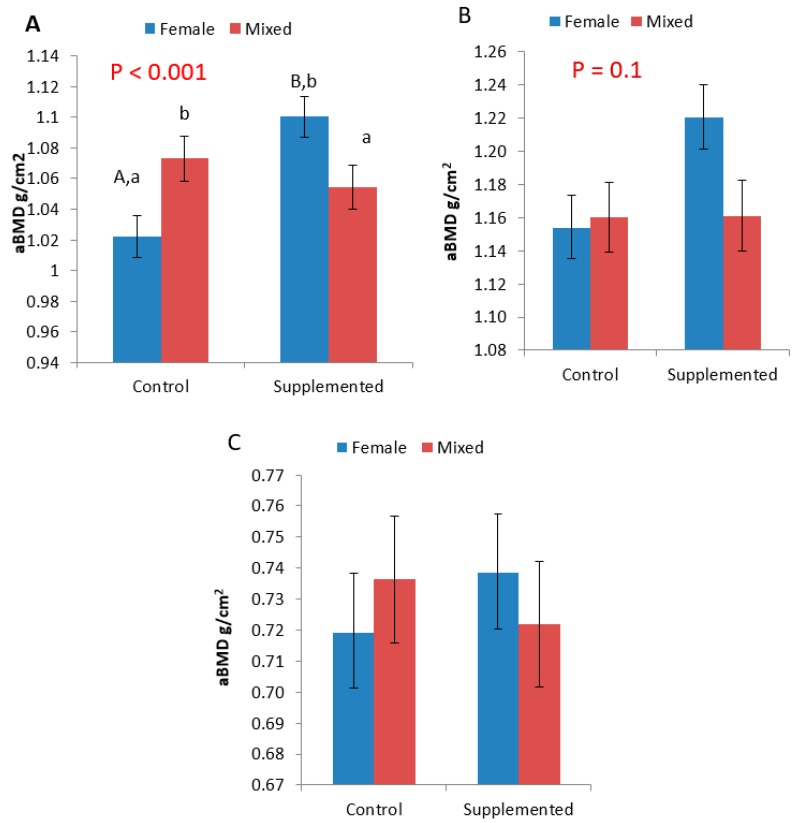
aBMD values for the (**A**) Humerus, (**B**) Radius/Ulna and (**C**) Metacarpal. The *P* values represent the interactive effect between group composition and diet. The lower-case letters (a,b) indicate a tendency for a difference of *P* < 0.1 between group composition and within dietary treatment, whereas upper-case (A,B) superscripts indicate a difference of *P* < 0.001 between dietary treatment and within group composition.

Gilts from the SUPP treatment had a greater aBMD (*P* < 0.05) in the HUM, and there was also an interaction between group composition and diet (*P* < 0.001; Figure 2). Gilts from the FEM treatment on the SUPP diet tended to have a higher aBMD than those from MIX groups on the SUPP diet (*P* = 0.1), and had a higher aBMD than FEM groups on the CON diet (*P* < 0.001). Within the CON treatment, there was a tendency for gilts from the MIX groups to have a greater aBMD than gilts from FEM groups (*P* = 0.06).

There was also a tendency for an effect of mineral supplementation in the RU, with SUPP gilts having greater aBMD (*P* = 0.1). There was no effect of either diet or group composition on the aBMD of the META (*P* > 0.05).

Numerically, we found that fractures were more common in MIX groups. However, the average aBMD in pigs without fractures FEM (0.99 ± 0.21) and MIX (0.98 ± 0.21) was lower than the aBMD of pigs with fractures; MIX (1.01 ± 0.22) and FEM (1.15 ± 0.36). As only five pigs had fractures (4.8% of all trial pigs), this could not be statistically analysed.

### 3.3. Cartilage Surface Lesions

**Figure 3:**
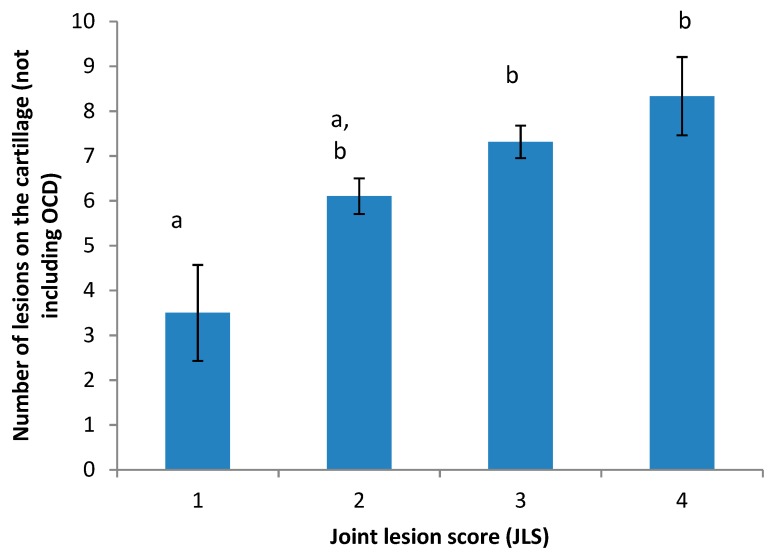
Comparison of the number of lesions on the joint cartillage relative to the overall joint lesion score (JLS) assigned. Levels of the JLS were 1 = normal, 2 = mild abnormality, 3 = moderate abnormality and 4 = severe abnormalitya, b indicates a significant difference in the number of lesions present, between JLS categories.

When comparing the two scoring systems (JLS *v’s* counting the lesions), there was an overall effect of JLS category (*P* < 0.01; Figure 3). Although the only significant difference between JLS categories was between 1 and 3, and 1 and 4 (*P* < 0.01 for both), there tended to be a difference between scores 1 and 2 (*P* = 0.1), 2 and 3 (*P* = 1), and 2 and 4 (*P* = 1).

There was no effect of either diet or group composition on JLS, or of group composition on the number of OCD lesions. However, gilts on the SUPP diet tended to have a lower number of OCD lesions than those on the CON diet (*P* = 0.09; Table 4). All gilts with OCD lesions in more than one location were on the CON diet.

With regard to the number of lesions on the cartilage, there was an effect of group composition; gilts reared in MIX groups had more disorders (thinnings, invaginations and overgrowths) present on the cartilage, when summed together (Table 5). Likewise, gilts on the CON diet, tended to have more disorders present than gilts on the SUPP diet (Table 5).

When considering only the HC, gilts reared in MIX groups also had higher total scores (Table 5). There were no other effects of either diet or group composition on the number of disorders. Overgrowths were too few to statistically analyse, but numerically, there were more instances in gilts on the CON diet than on the SUPP diet (Table 5). Fractures of the HC were found in five gilts (4.9% of all examined gilts). Four of these were gilts reared in MIX groups (Table 4).

## 4. Discussion

This study investigated the effect of a mineral supplemented diet and group composition on the limb health of replacement gilts with the ultimate aim of improving gilt longevity and welfare and reducing culling for lameness. Supplementation of the gilts’ diet during rearing with the trace minerals copper, zinc and manganese led to increased bone mineral density, while rearing gilts in female-only pens reduced the number of joint lesions associated with cartilage degradation, and there were numerically fewer incidences of fractures in the humeral condyle (HC) joint. Female-only rearing and mineral supplementation combined resulted in an important beneficial effect on bone mineral density of the humerus.

Several authors found improvements in locomotory ability in gilts on mineral supplemented and/or specially formulated developer diets with similar levels of copper, zinc and manganese, as employed in this trial [7,8,24]. In contrast, we observed no beneficial effect of mineral supplementation on locomotion scores. Indeed, there was a tendency at day 148.3 ± 8.0 (the last locomotion scoring point before the gilts were slaughtered at d196.3 ± 0.5) for gilts in the mineral supplemented groups to have higher locomotion scores. However, Quinn [8] included the mineral supplement in a diet formulated for fat rather than lean meat deposition (i.e., a developer diet). The diet was restricted fed, with the aim of slowing growth rates, which necessitated housing the gilts individually. This was compared to a standard finisher diet. In the other study by Quinn [7], un-supplemented finisher and gestating sow diets were compared to a supplemented developer diet which also had a higher energy to lysine ratio compared to the other two diets, and terminal line gilts were used. Thus, although similar to the current study, the mineral supplemented diets also had other critical differences to a finisher diet which could have promoted limb health. Faba [24] reared gilts on four diets; control, mineral supplemented (copper, zinc and manganese), methionine supplemented and a methionine plus mineral supplemented diet. Additionally, gilts were reared in groups of 10, which is a lower number than our study. In the current study all the gilts were on a standard finisher diet and in contrast to Quinn [7] and Faba [24], half of them were kept in mixed-sex groups. Clearly, our findings indicate that supplementation of a finisher diet with minerals associated with bone/limb health alone is not enough to influence locomotory ability. On the other hand, locomotory ability was generally very good across all treatments and no lameness was detected in any of the trial animals.

In line with Quinn [7,8] we found a beneficial effect of mineral supplementation on bone mineral density. In the study by Quinn [8], the effect was only numerical, which could be explained by the later stage at which the supplementation was applied (i.e., d130 vs. d117.5 ± 0.6), and the fact that the animals were individually housed, therefore, restricted in their movements. In the other study by Quinn, which applied the supplementation at a similar age to the current work (~112d of age), and in which the animals were managed in groups, there was a significant difference in BMD in developer diet-fed gilts compared to finisher diet-fed gilts [7].

Mineral supplementation had the greatest impact on the bone mineral density of the humerus. Bio-mechanically, the front limbs carry more load [25], have higher peak forces when walking, and have a longer stance phase [26].The humerus is the biggest bone in the front limb and it is possible that this bone bears a greater proportion of weight relative to the radius and ulna, where two bones are involved, and the metacarpals, where there are multiple digits. The lack of an effect of mineral supplementation on the other bones examined could also partially be explained by the relative difficulty in scanning these bones relative to the humerus. The latter bone, because of its larger size, was very easily positioned for obtaining a clear scan by the DEXA scanner. In contrast, the radius and ulna may overlap, which can influence the aBMD reading [27]. Schenck [27] separated the fibula and tibula for DEXA scans for better accuracy of BMD readings. The metacarpal was the most difficult bone to scan, not only because of the risk of overlap between the metacarpals, but also because of the difficulty in positioning it at a consistently stable angle on the table. However, most studies used the metacarpal for analysis of aBMD [28,29,30], as it is believed to be a sensitive bone to mineral changes in diets [31,32,33,34]. Garg [35] stated that the femur is used to analyse BMD in humans—which when compared with all three bones in our study is most similar to the humerus. In a sow study, Crenshaw [36] used the humerus to determine bone mineral content by bone ash mineral analysis.

Interestingly, and in contrast to our hypothesis, when fed the standard finisher diet, gilts from the mixed-sex groups tended to have a greater BMD than the gilts from female-only groups. Weight bearing exercise was associated with improvements to BMD in humans [37,38] and in fact, there is evidence that increased exercise in pigs also increases it [39]. In another study, it was found that osteochondrosis risk was reduced by regular but limited exercise in young foals [40]. In fact, in a sister paper to this, we observed increased levels of aggression and mounting in mixed-sex pens, as well as play behaviour ([41] *in preparation*) so increased activity in general. Stavrakakis [42] stated that bone remodeling allows for the ability to adapt according to the environment and the loading the animal is exposed to. Therefore, a pig experiencing more mechanical stress could increase bone mass accordingly. However, overloading and the stress of heavier bodyweight can cause a disturbance in metaphyseal blood flow, which can cause joint lesions on immature cartilage [43]. Additionally, this mechanical pressure can cause a disturbance in blood flow at bone-cartilage junctions, causing osteochondrosis lesion prevalence [44].

We compared both systems of scoring joint lesions so that we could confirm that the newer system which we developed (counting all lesions) produced similar data, with the same direction and magnitude of differences between scores as the previously published version (JLS; [8]). The newer scoring system was developed in an attempt to improve the objectivity of the scoring system and also so that we could determine whether any differences between treatments were due to particular pathologies. Nevertheless, it was important that we compare both so that the results from our study could be discussed in the context of previously published work. Data from both systems corresponded with one another, in that, as the JLS score increased, so did the number of lesions on the cartilage. Moreover, counting the lesions enabled us to discern differences between treatment groups in several measures, whereas the JLS system did not. As there were only four joints with a score of 1, and six with a score of 4, it is likely that with a larger sample size, some of these differences may have been significant. Thus, for this type of study, counting the different types of lesions present may improve the ability of the researcher to detect treatment effects.

Female-only rearing also had a beneficial effect on the number of joint lesions observed. This is consistent with our hypothesis, as in mixed-sex groups, females were subjected to potential trauma caused by sexual mounting and aggression performed by entire males [9,10,11]. This was exacerbated by housing on fully slatted floors [45,46]. Indeed, four of the five gilts with fractures in the humeral condyle at slaughter were reared with entire males in mixed-sex groups. This equates to 5% of all the gilts on trial and does not differ greatly from the 10% prevalence of fractures in cull sows [47,48]. As fractures occur during falls, fights and attempts to free a leg [49,50,51], trauma associated with the behaviour of entire males cannot be discounted as a possible cause. While the injuries may have occurred during transportation or in the slaughterhouse, the finding still suggests an increased susceptibility for breakage associated with mixed-sex rearing, possibly because of pre-existing damage.

The presence of fractures in five of the gilts, as well as the high prevalence (100%) of joint lesions and the fact that over a quarter of the animals had OCD lesions irrespective of treatment, raises concern for gilt welfare. This was complicated by the lack of an association between locomotion scores and joint lesions/OCD as the possibility of detecting such problems is reduced. Bone strength and likeliness of fracture is related to BMD [52]. Surprisingly, in this study, the average BMD in fracture pigs was higher than in non-fracture pigs. As the fracture pigs only account for 5% of trial pigs, it is a matter for future research. Indeed, there is also an association between OCD and physical injuries, such as joint lesions and bone fractures [53,54]. Certainly, earlier identification could allow gilts that portray signs of leg weakness to be removed from the replacement herd [55]. The lack of an association is in accordance with unpublished work by Quinn in which there was no association between lameness (locomotion scoring) and OCD or bone mineral density (Quinn, [7]). Quinn [8] found that there was no correlation between locomotion and aBMD. However, there tended to be a correlation between lesions on the HC and locomotion scores.

Numerous studies have reported no association between OCD and lameness/leg weakness [56,57,58,59,60] and the majority of studies supporting the association involved sows that were specifically culled for lameness [2,3,47,48,61]. Given that treatment effects were detected in bone mineral density and cartilage degradation scores, the findings regarding locomotory ability suggest that either the locomotion scoring system was not sensitive enough to detect any difference, or differences in BMD and OCD are not reflected in locomotory ability. Indeed, the relationship between lameness and OCD is poorly understood [62,63].

Nevertheless, it is possible that a more sensitive lameness/locomotory scoring system could help identify gilts suffering from OCD. Although locomotion scoring is of limited reliability [49,64,65,66] if it was possible to identify features of locomotion which portray a long-term locomotory ability and assess different locomotory patterns that are associated with lameness, a more objective, reliable and accurate scoring system could be created [42]. The scoring system employed in the current study attempts to identify an affected limb [66]. However, as OCD occurs bilaterally in the elbow joints [43,54,58,67,68], the pain is likely equalised across both limbs and therefore, it might be very difficult for a pig to reduce the weight on one of them. A method of locomotion scoring which takes into account different aspects of limb and body movements may be more useful in detecting subtle differences associated with limb or joint pain. The development of a new locomotion scoring protocol for different age groups would also be very useful.

## 5. Conclusions

This study found benefit to replacement gilts of feeding an increased level of the trace minerals copper, zinc and manganese over those recommended for finisher pigs, on several measures of limb condition. This was the case even though these differences were not detectable using a locomotion scoring system. The benefits of supplementing these minerals could lead to potential improvements in the lifetime performance of replacement gilts and the longevity of sows. Separating replacement gilts from finishing stock, particularly entire males, can significantly reduce incidences and the severity of joint lesions and OCD. Thus, there is a clear indication that female-only pens and a mineral supplemented diet for replacement gilts can have benefits for limb heath and their welfare.

## Figures and Tables

**Table 1:** Details of the finisher (CON; fed to all pigs) and supplemented (SUPP; from day 117.5 ± 0.6/week 16.8 ± 0.1 of age) diets which were used in the study.

Ingredients	CON	SUPP*
Barley	50	50
Wheat	33.50	33.38
Soybean (47%CP)	12	12
Soya oil	1	1
Lysine HCl	0.4	0.4
dl-Methionine	0.1	0.1
l-Threonine	0.12	0.12
Premix ^a^	0.1	0.1
Availa®Sow ^b^	0	0.1
Phytase	0	0
Salt feed grade	0.5	0.5
Di-Calcium phosphate	1.3	1.3
Limestone flour	1	1
	100.02	100.00
Chemical composition		
Dry matter	89.8	89.8
Crude protein	15.56	15.56
Crude Fibre	3.74	3.77
Total oil	5.06	5.06
Ash	4.48	4.48
Lysine	0.969	0.969
Threonine	0.639	0.639
Methionine	0.337	0.337
Methionine and cysteine	0.639	0.639
Tryptophan	0.182	0.182
Calcium	0.779	0.779
Phosphorous	0.609	0.608
Digestible phosphorus	0.280	0.280
Digestible energy (MJ of DE/kg)d	13.50	13.49

^a^ Premix provided per kilogram of diet Cu, 15 mg; Fe, 24 mg; Mn, 0 mg; Zn, 30 mg; I, 0.15 mg; Se, 0.4 mg. ^b^ Availa®Sow provided per kilogram of complete diet, Zn, 50 mg; Mn, 20 mg; Cu, 10 mg. * Supplemented with Availa®Sow (Zinpro Corp).

**Table 2:** Mineral inclusion rates in the diets of control (fed to all pigs) and supplemented (SUPP; from day 117.5 ± 0.6/16.8 ± 0.1 wks of age) finisher pigs. Values are expressed as mg/kg (i.e., parts per million, and as a % of NRC recommendations for gestating and lactating sows.

	NRC (mg/kg) ^1^	Control (mg/kg)	SUPP (mg/kg)	Control % ^2^	SUPP % ^2^
Mn	25	25.1	51.45	101%	206%
Zn	100	55.6	122.29	56%	122%
Cu	10	4.5	17.89	45%	179%

^1^ NRC gestating and lactating sow requirements. ^2^ Values in the control and mineral supplemented diet as a percentage of the NRC recommendations.

**Table 3:** Scoring system for locomotory ability (adapted from *Calderon-Diaz* [23]).

Score	Description
0	Even strides. Pig is able to accelerate and change direction rapidly
1	Pig appears stiff. Abnormal stride, which isn’t easily identified. Movements no longer fluid but pig still able to accelerate and change direction rapidly. Caudal swagger evident.
2	Uneven stride. Sensitivity while walking detected on at least one limb. Pig able to accelerate and change direction. Caudal swagger evident
3	Uneven stride, with a stagger. Minimum-weight bearing on affected limb. Slow to move. Obviously lame even to the untrained observer
4	Pig may not place affected limb on floor
5	Does not move

**Table 4:** Cartilage damage and OCD presence in the joints of gilts reared in either mixed sex (MIX) or female-only (FEM) groups and on a standard finisher diet (CON) or on a CON diet supplemented with minerals (SUPP).

	Diet		Group Composition	
	CON	SUPP	MIX ^1^	FEM
No. animals	51	50	45	56
Cartilage damage				
No. animals without OCD lesions	35	39	31	43
Sum of cartilage scores	88	103	82	109
OCD lesions				
No. gilts with 1 area ^2^	10	11	10	11
No. gilts with 2 areas ^2^	3	0	1	2
No. gilts with 3 areas ^2^	3	0	3	0
Total no. gilts with OCD	16	11	14	13
No. gilts with fractures	2	3	4	1

^1^ 6 males and 6 females per group ^2^ Data are provided as the number of occurrences of OCD lesions. 1 = OCD lesion present on HC, 2 = Two OCD lesions present on the HC, 3 = Two OCD lesions present on the HC and one present on the TN.

**Table 5:** The number of instances of thinning, invagination, and overgrowth of the cartilage in the joints of gilts reared in either mixed sex (MIX) or female-only (FEM) groups and on a standard finisher diet (CON) or on a CON diet supplemented with minerals (SUPP).

	Diet			Group Composition			Interactive Effect
	CON	SUPP	*P*-value	MIX ^1^	FEM	*P*-value	*P*-value
No. animals	51	51	.	46	56	.	.
Total * ^2^	7.21 ± 0.36	6.42 ± 0.36	0.12	7.39 ± 0.38	6.24 ± 0.34	0.03	NS
Thinnings ^3^	4 (2–5)	4 (2–5)	0.37	4 (3–5)	3.5 (2–5)	0.45	0.13
Invagination ^3^	2 (2–3)	3 (2–4)	0.60	3 (2–4)	2 (1–3)	0.27	NS
Overgrowth ^4^	17	8	.	13	12	.	NS
HC total ^2^	5.94 ± 0.39	5.48 ± 0.31	0.28	6.23 ± 0.33	5.19 ± 0.30	0.02	NS
HC thinnings ^3^	3 (2–4)	3 (2–4)	0.72	4 (2–5)	3 (2–4)	0.20	0.14
HC invagination ^3^	2 (1–3)	2 (1–3)	0.47	3 (2–3.5)	2 (1–3)	0.26	NS
HC overgrowth ^4^	12	7	.	10	9	.	NS
TN total ^2^	1.26 ± 0.16	0.94 ± 0.15	0.16	1.14 ± 0.16	1.06 ± 0.15	0.76	NS
TN thinnings ^3^	1 (0–1)	1 (0–1)	0.44	1 (0–1)	1 (0–1)	0.80	NS
TN invagination ^3^	0 (0–1)	0 (0–0)	0.52	0 (0–1)	0 (0–0.5)	0.79	NS
TN overgrowth ^4^	5	1	.	3	3	.	NS

^1^6 males and 6 females per group ^2^Analysis carried out using linear mixed models. Results are provided as least square means and standard errors. ^3^Analysis carried out using generalised mixed models. Results are presented as median and interquartile ranges. ^4^Due to the low incidence number, no analysis was carried out. Data are provided as the sum of the number of each disorder. * Sum of the number of all disorders (thinnings, invaginations and overgrowths).

## Supplementary Materials

The following are available online at https://www.mdpi.com/2076-2615/9/9/637/s1. Table S1: Rules applied for statistical purposes using SAS 9.4 where numbers of scores at the extreme ends of scoring scales were too small to statistically analyses.
