# Supplementary material for: The Effect of Group Composition and Mineral Supplementation during Rearing on Measures of Cartilage Condition and Bone Mineral Density in Replacement Gilts
Source: Animals (Basel). 2019 Aug 30;9(9):637. doi: 10.3390/ani9090637 (PMC6770507; doi:10.3390/ani9090637)
Supplement: Supplementary file 1 [file animals-09-00637-s001.pdf]

Table S1. Rules applied for statistical purposes using SAS 9.4 where numbers of scores at the extreme ends of scoring scales were too small to statistically analyse.

**Disorder**	**Rule**
HC thinnings	Thinnings >5 = 5;
HC invaginations	Invagination >4 = 4;
	Invagination <1 = 1;
TN total	Inner >3 = 3;
Inner thinning	Inner thinning >1 = 1;
Inner invagination	Inner invagination >1 = 1;
	Inner invagination <1 = 0;
	Inner invagination = 1 = 1;
Overgrowths	Overgrowth <1 then delete;
